# Locust Bacterial Symbionts: An Update

**DOI:** 10.3390/insects11100655

**Published:** 2020-09-24

**Authors:** Omer Lavy, Uri Gophna, Eran Gefen, Amir Ayali

**Affiliations:** 1 School of Zoology, Tel Aviv University, Tel Aviv 6997801, Israel; 2 School of Molecular Cell Biology and Biotechnology, Tel Aviv University, Tel Aviv 6997801, Israel; urigo@tauex.tau.ac.il; 3 Department of Biology, University of Haifa–Oranim, Kiryat Tivon 3600600, Israel; gefene@research.haifa.ac.il

**Keywords:** locust, bacteria, locust symbionts, desert locust, migratory locust

## Abstract

**Simple Summary:**

Locust plagues have been devastating vegetation and agriculture since ancient times. The tendency of locusts to form huge swarms and migrate long distances is a hallmark of the locust phenomenon. The ecological and economic importance of locust plagues has attracted much research into different aspects of the natural history of these insects. One of the many investigated aspects of the locust phenomenon is that of their interaction with the bacteria they harbor in and on their body. These locust-associated bacteria have been shown to affect certain physiological traits (such as immunity and swarm cohesion), as well as possibly affecting and being affected by different factors in the locust biology. Here, we review the current understanding of the locust–bacteria interaction. We focus on identifying the bacterial strains and their locations within the insects; the role of the bacteria and their importance to their host’s life; the mechanism of transmitting important bacteria across locust generations; and more. Finally, we offer some new perspectives and research directions that could broaden our understanding of the locust-associated bacteria and their role in locust outbreaks.

**Abstract:**

As one of the world’s most infamous agricultural pests, locusts have been subjected to many in-depth studies. Their ability at one end of their behavioral spectrum to live as solitary individuals under specific conditions, and at the other end of the spectrum to form swarms of biblical scale, has placed them at the focus of vast research efforts. One important aspect of locust ecology is that of their interactions with the bacteria that reside in and on them. Although this aspect of locust ecology has been little studied relative to the mainstream locust research, these bacteria have been shown both to affect locust immunity and to participate in maintaining swarm integrity through the secretion of attractant volatiles. The interaction between locusts and their bacteria seems, however, to be bi-directional, with the bacteria themselves, as recently shown, being influenced by their host’s swarming tendencies. This seems to be a consequence of the bacterial composition in the locust’s gut, reproductive organs, and integument undergoing change with the change in their host’s behavior. In this review we describe the current state of knowledge of the locust–bacteria interactions (data exists mainly for the desert and the migratory locusts), as well as highlighting some newly-gained understanding; and offer perspectives for future research.

## 1. Introduction

Locusts are short-horned grasshoppers (Orthoptera) belonging to the family Acrididae. A hallmark of locust biology is their density-dependent phase-plasticity, in which individuals will express vastly different behavioral, morphological, and physiological phenotypes according to the different population density levels they experience [1,2,3]. Locusts are probably best known, however, for their devastating potential to cause immense damage to natural vegetation and crops, as they are able to aggregate and migrate in swarms so large and destructive that they are commonly termed a plague. Consequently, locusts have been the focus of extensive research efforts, mostly seeking to better understand the mechanisms underlying the locust swarming phenomenon.

Although not part of the major emphasis of previous locust research, data concerning the characterization of locusts’ bacterial symbionts were published already in the late 1950s [4,5,6]; and since then a multitude of important observations have accumulated regarding the nature of locust–bacteria interactions [7,8,9,10,11,12,13,14,15,16,17,18]. Some of these findings were already summarized, nearly 20 years ago, by Dillon and Charnley [10], but ever since their extensive review, many new data have been collected, and vast improvements in sequencing technology have allowed researchers to ask and answer questions previously beyond our reach. The wealth of new findings in recent years calls for an update, incorporating the essential details of our current knowledge of locust-bacteria associations.

In this concise review, we provide an updated summary of the nature of locust bacterial symbionts and their distribution in the host, as well as hypothesized effects on the locust biology and life history. We mainly focus on the desert locust (*Schistocerca gregaria*) and the migratory locust (*Locusta migratoria*), which most of the studies conducted to date have explored. However, the main ideas presented will be largely applicable to other locust species, albeit perhaps with different bacterial players.

## 2. Locust Bacterial Symbionts and Their Location

Similar to most vertebrates and invertebrates, locusts harbor bacterial cells both within their internal body environment and externally on their cuticle [19,20]. Below we briefly present major data that have been gathered concerning the bacterial symbionts present in the locust digestive tract, as well as some more recent reports describing the bacterial symbionts in other parts of the locust’s body.

### 2.1. The Digestive Tract

The bacterial composition of the locust gut has been thoroughly described for the desert locust [7,10,11,12,15,18,21]. Some research has also been conducted on several other locust species, including the migratory locust [14,22,23], the brown locust (*Locustana pardalina*), the Moroccan locust (*Dociostaurus maroccanus*), and the Italian locust (*Calliptamus italicus*) [13].

**Figure 1:**
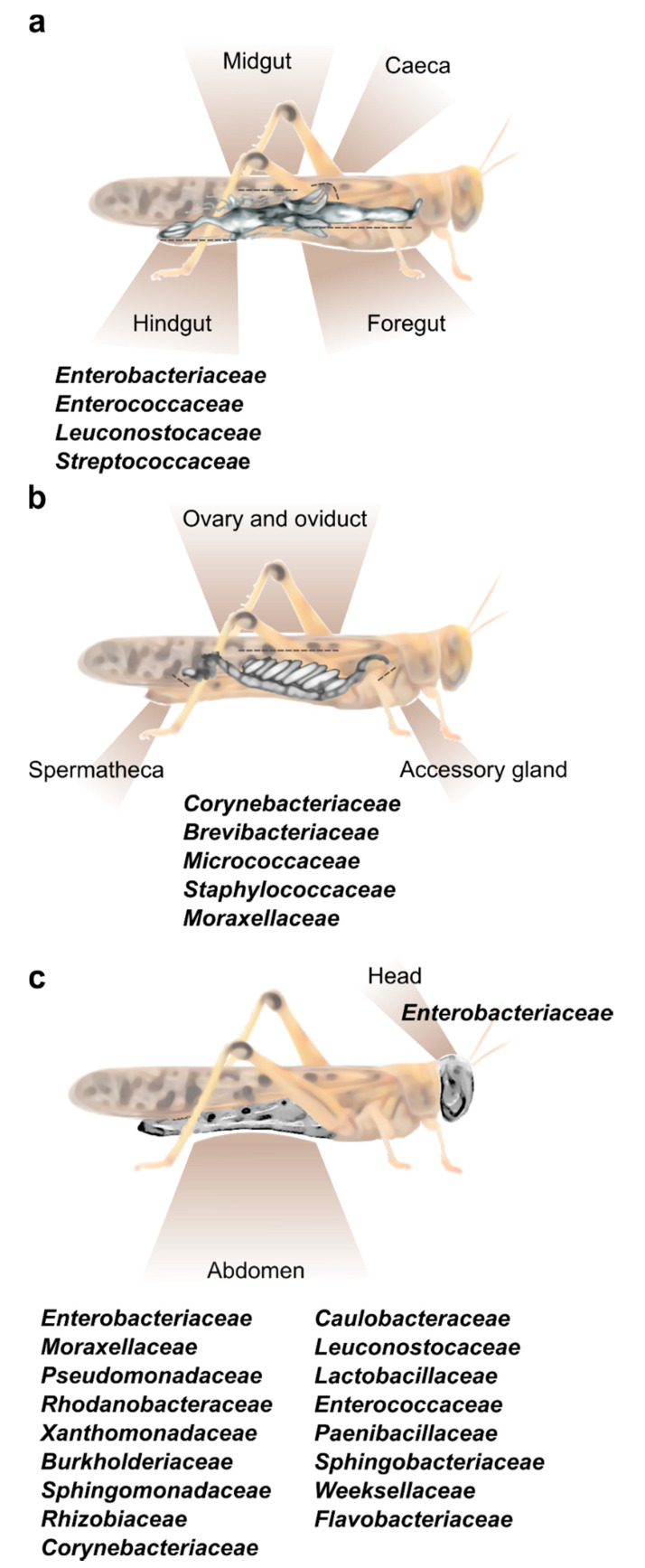
**The main bacterial families known to inhabit different body parts of the desert locust.** (**a**) A depiction of the locust’s alimentary canal and the common bacterial families residing within the hindgut. Foregut and hindgut (including caeca) do not host a constant fraction of specific bacterial strains [11,15]. (**b**) A depiction of the female locust reproductive system divided into three main sections, all harboring the same core bacterial families [16]. (**c**) External bacteria found on the head and abdomen cuticle of the desert locust [18,26].

The composition of the bacterial community in any insect alimentary tract is largely affected by its specific anatomy. The locust gut is relatively simple and consists of three main parts: foregut, midgut, and hindgut (Figure 1a). In brief, the cuticle-lined foregut is where the ingested food is mixed with salivary and regurgitated enzymes to initiate the food breakdown process; the food is then transferred to the midgut, where the digestion is completed and nutrients (mainly protein and carbohydrates derivatives, i.e., amino acids and monosaccharides) are absorbed, while a peritrophic membrane envelops the digested food. Six caeca invaginations are connected to the anterior part of the midgut, each opens individually into the alimentary canal (Figure 1a). The main role of the hindgut is the absorption of water, salts, amino acids, and small metabolites. Six large lobes, called “rectal pads”, maximize the surface area of the hindgut to enable efficient absorption [1,24,25].

Hunt and Charnley [8] used microscopy and cultivation-dependent methods to identify the bacterial residents in different parts of the *S. gregaria* alimentary canal. They observed that the bacterial load was small in the anterior parts of the digestive system, and that this load increased significantly in the posterior parts. They further reported that no bacterial cells were found on the lining of the foregut or on the epithelial cells of the midgut (including caeca), where bacteria were limited to the lumen and to the inner section of the peritrophic membrane. Furthermore, they noted that the bacteria found in the foregut were roughly similar to those found in the locust’s food, while the hindgut contained a high load of *Enterobacteriaceae* and *Streptococcaceae*. These important findings served to focus subsequent studies on those bacteria residing in the locust hindgut.

Several reports on the hindgut bacteria of *S. gregaria* have revealed a relatively simple bacterial composition, with a consistent presence of members of the families *Enterobacteriaceae* (mostly the genera *Enterobacter* and *Klebsiella*) and *Enterocococeaea* reviewed in [10] and described in [15,16,18,21,26]. *Enterobacteriaceae* and *Enterocococeae* members were also found in the hindgut of healthy *L. migratoria* [14], as well as in the gut of field-caught Italian locusts, Moroccan locusts and brown locusts [13].

Based on the fact that desert locusts were shown to be able to live and reproduce without their gut bacterial symbionts, at least under laboratory conditions [27], it is accepted that locusts have developed a tight but not obligatory association with these mutualistic bacteria. Yet, some bacteria seem to play an important role in the fitness of locusts by conferring colonization resistance and by means of their volatile secretions (to be discussed below). This tight but not obligatory interaction may be routed in the function that these bacteria execute for their host. If more than one bacteria can produce the same essential product for the locust, this will push towards facultative, rather than obligatory, locust–bacteria interaction [28]. For example, if both *Enterobacter* and *Klebsiella* can produce cohesion promoting volatiles and to confer gut-colonization resistance, harboring one strain or the other will not affect the locust’s fitness, thus promoting the non-obligatory interaction, where the gut niche is colonized by the first suitable bacteria to be ingested by the locust.

The genus *Weissella* (Firmicutes: family *Leuconostocaceae*), was repeatedly found in the gut of *L. migratoria* [14,22,23], as well as following high-throughput sequencing of the desert locust’s gut microbiota [15,18]. The fact that this genus was absent from previous reports of the bacterial composition of locusts, may be attributed to difficulties in growing it in culture, which can be circumvented by advanced sequencing methods. Thus, it is highly plausible that additional important locust symbionts have not been cultivated to date and thus their effect on the host may be currently underappreciated.

### 2.2. The Female Reproductive System

As already noted, the organ at the spotlight of locust–bacteria research to date has been almost exclusively the gut. However, a recent report by Lavy et al. [16] focused on the reproductive system of the female desert locust and revealed the unique bacterial composition of different organs in this system. That study divided the female gonads into three main parts—accessory glands, ovaries, and spermatheca (Figure 1b), and used high throughput amplicon sequencing to analyze their bacterial composition. This resulted in the observation that the reproductive organs contained a consistent fraction of *Corynebacterium*, *Brevibacterium* and *Micrococcus* (Actinobacteria), along with *Staphylococcus* (Firmicutes) and *Acinetobacter* (Gammaproteobacteria) (Figure 1b). This unique bacterial composition of the female reproductive organs did not seem to be affected by mating, or by oviposition (during which the female extends her abdomen into the ground and oviposits at a depth of ~10 cm; [29]). The presence of the genus *Brevibacterium* in locusts had been reported only once prior to the aforementioned study: Bucher and Stephens [5] used a whole-body homogenate to examine the locust’s gut bacterial composition. Some bacteria isolated by them belonged to the genus *Brevibacterium*, leading these authors to consider it to be a gut-residing bacterium. However, later research, employing an isolated-gut approach, failed to find this bacterium in the locust gut. It is, therefore, possible that the origin of *Brevibacterium*, as described by Bucher and Stephens [5], was the locust reproductive system, and it was mistakenly described as a gut bacterium due to the experimental procedure.

### 2.3. The Cuticle

Our group recently sampled the abdominal cuticle of laboratory-reared *S. gregaria* in order to characterize its bacterial composition, as part of a larger study examining also the bacterial content of the fecal pellets of the same individuals [18]. It was then observed that the locust cuticle harbors a large fraction of what might be gut-associated bacteria, such as *Weissella* and other members of Firmicutes and Proteobacteria, which were also found in the feces (and which could have been amplified due to the crowded conditions in the cage). However, the bacteria residing on the locust integument also featured members of the phyla Bacteroidetes (*Flavobacteriaceae* and *Weeksellaceae*) and Actinobacteria (*Corynebacteriaceae*), which were unique to the external cuticle and were not present in the locust feces [18]. In another recent study [26], cultivation-dependent methods identified a species of Salmonella (Proteobacteria) externally on the head and body of *S. gregaria*, in agreement with our own observations noted above (Figure 1c).

## 3. Locust–Bacteria Mutualism

Bacterial symbionts have been repeatedly shown to play an important role in the biology of diverse insect species [30,31,32,33]. In a number of studies, bacteria were reported to augment aphid resistance to various stressors [31], to protect the gardens from Attine ants from pathogens [34], to protect wasp-larvae from potential pathogens [35], and more. The symbiotic nature of the locust–bacteria interaction has also been studied extensively, mostly in the desert locust and to some extent also in the migratory locust [10,11,12,13,14,15,16,17,18,21,22,23].

As noted above, the bacterial composition of the desert locust foregut was found to be roughly similar to that of the food ingested by the locusts. The bacterial community of the hindgut, however, differed from that in the food and the foregut, demonstrating a higher prevalence of *Enterobacteriaceae* cells [8]. This finding and the multiple subsequent confirmations of the stable presence of *Enterobacteriaceae* species in the locust hindgut suggested a selective basis for the interaction of *S. gregria* and bacteria from the *Enterobacteriaceae* family. Dillon and Charnley [10] hypothesized that the neutral pH of the hindgut (along with the presence of organic acids and plant remains) supports the proliferation of *Enterobacteriaceae*. This hypothesis was confirmed to some extent in *L. migratoria* by Shi et al. [14], who observed that acidification of the locust’s hindgut by entomopathogenic fungus reduced the bacterial colony-forming units (CFU) isolated from this organ. Those authors also demonstrated experimentally, by mimicking the acidification effect of the fungus on the hindgut, that the growth of locust-isolated *Enterobacter* sp. (*Enterobacteriaceae*) was almost completely inhibited when pH dropped from 6.3 (as in the hindgut of healthy *L. migratoria*) to 5.6 (reflecting the fungus impact). The growth of locust-isolated *Weissella*, *Enterococcus*, and *Microbacterium* was inhibited by the low pH of 5.6, though not to the same extent as the tested *Enterobacter*. Consequently, although the locusts had clearly not developed obligatory interactions with any specific bacteria, the specific conditions in their hindgut seem to have been selected for the proliferation of certain bacterial groups, including the *Enterobacteriaceae* family.

Native bacteria of the desert locust’s hindgut have been shown to augment host immunity through colonization resistance (CR), thereby making it less susceptible to pathogenic infections [12]. This is largely due to the bacterial synthesis of antifungal and antimicrobial phenolic compounds [35,36,37]. The antimicrobial nature of these compounds not only protects the locust from the establishment of pathogens, but also probably contributes to the selective conditions in the locust hindgut, creating favorable conditions for the anti-microbial tolerant native bacteria of the locust gut [10,12].

Two of the reported bacteria-secreted phenolic compounds, phenol and guaiacol, were also suggested as a dominant fraction of a cohesion pheromone, considered to help in maintaining the integrity of *S. gregaria* swarms [11,38]. Both phenol and guaiacol were shown to be produced by three bacterial species in the locust hindgut: *Pantoea agglomerans*, *Klebsiella pneumoniae pneumoniae*, and *Enterobacter cloacae* (all from the *Enterobacteriaceae* family), when incubated on axenic (i.e., bacteria-free) locust fecal pellets. The precursor for these phenolic compounds in the gut is the lignin-derived vanillic acid present in the locust’s food. *Serratia marcescens* a member of the *Enterobacteriaceae* which is not a regular inhabitant of the locust gut, did not produce the same phenolic volatiles [11], reinforcing the hypothesis of a mutual symbiosis between the host and specific bacterial species. Additional information on the phenolic compounds produced by the desert locust’s gut bacteria can be found in the excellent review by Dillon and Charnley [10].

## 4. Transgenerational Transmission of Bacterial Symbionts

Desert locusts are highly polyphagous, and therefore have not developed a known obligatory symbiosis with any bacterial species, unlike the obligate–symbiosis interactions known in many diet-restricted insects [1,10,33,39]. Nevertheless, as noted above, it seems that locusts have formed a tight and constant interaction with some bacterial species (i.e., species of the *Enterobacteriaceae* family). Although the benefits of this symbiotic association are clear, it is still not completely understood how this interaction is maintained across locust generations. Based on the findings of Hunt and Charnley [8] and the successful rearing of axenic locusts, Dillon and Charnley [10] suggested that the source of locust bacterial symbionts is the ingested food, and that the locust gut selects for the favorable symbionts. The issue of where gut symbionts originate from has been revisited in a recent study by our group. In a series of controlled experiments, we have shown that female locusts share their gut-derived bacterial amplicon sequences variants (ASVs) of *Enterobacter*, *Klebsiella* and *Corynebacterium* with their offspring. The inoculation vector ensuring the successful transgenerational transmission of bacterial symbionts was found to be the foam deposited above the egg pod following oviposition (through which the larvae climb upon hatching). This was supported by an experiment demonstrating that females inoculated the foam plug with viable *K. pneumonia* marked with an antibiotic resistance marker [17]. This study further presented the foam plug as a selective medium, favoring some bacterial species and inhibiting others, probably due to immune-related proteins such as lysozyme, which were found to be present in the foam.

Based on the current knowledge, therefore, we suggest that locusts acquire their bacterial symbionts through both food intake and parental inoculation. Swarms of gregarious locusts perform multigenerational migrations across vast distances, resulting in the locusts encountering different food plants and the females ovipositing in unfamiliar (soil and food-wise) territories [29,40]. This mixed inoculation model ensures that the offspring will harbor the beneficial symbionts. The vertical transmission of *K. pneumonia*—a known nitrogen-fixing bacterium [41]—is especially interesting in the context of locust swarming and migration, since it may serve to compensate for poor/insufficient nutrition that locusts may encounter during long-distance migration.

## 5. Bacterial Symbionts and Density-Dependent Phase Polyphenism

**Figure 2:**
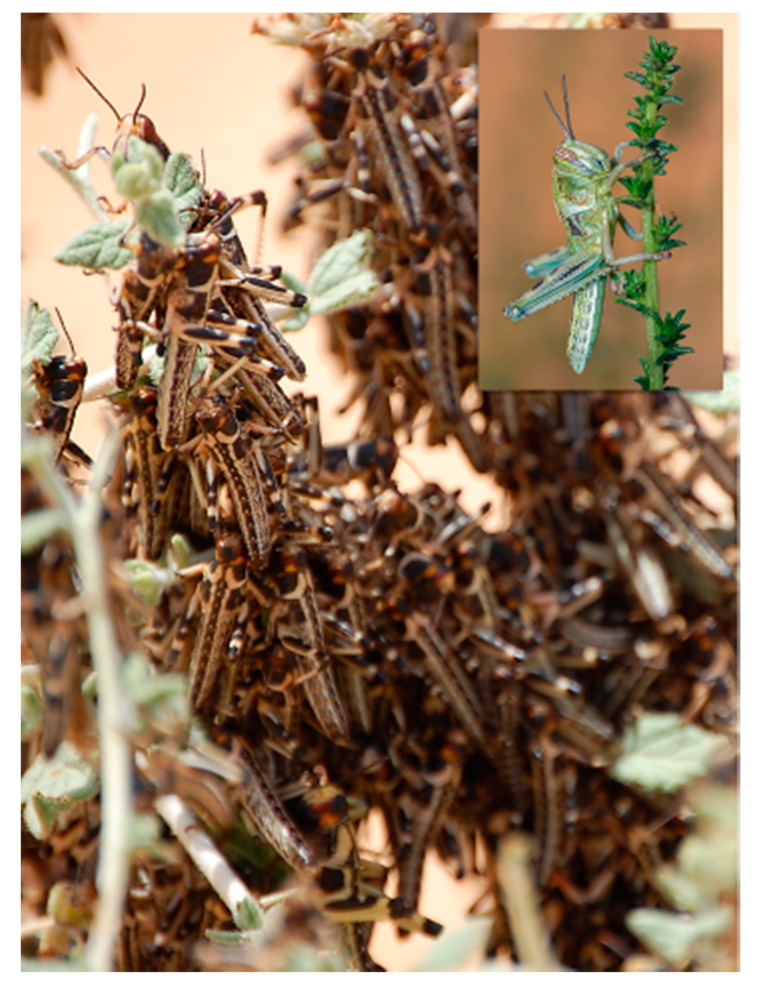
**Locust density-dependent phase polyphenism.** Gregarious locusts demonstrating extremely high density while perching on the vegetation (IV instar nymphs). Inset shows a solitary nymph. Photo credit: Amir Ayali, Gil Wizen (Inset).

Locusts are known to express two extreme phenotypes [1,2,3], Figure 2. When at high density, locusts tend to aggregate and migrate in swarms, consuming vast amounts of vegetation, and are thus considered to be a major agricultural pest. At low density, however, locusts tend to behave cryptically. They are relatively sedentary, repelled by conspecifics are at too low a density to cause severe damage to crops. This remarkable example of phenotype plasticity is known as density-dependent phase polyphenism, and has been amply investigated for reviews see: [1,2,3]. The seminal findings concerning the bacterial origin of *S. gregaria* cohesion pheromone components [11], provided a strong indication that bacterial symbionts may play a role in locust phase polyphenism.

Dillon et al. [13] used the denaturing gradient gel electrophoresis (DGGE) technique and 16S rRNA sequencing to explore and compare the bacterial composition of gregarious, transient (intermediate phase), and solitary field-caught brown locusts. They showed that the gut of gregarious and transient individuals contained a more complex bacterial composition compared to the solitary locust gut bacterial microbiota. Discussing these results, the authors raised the possibility that locusts of different phases regulate their microbiota differently. They hypothesized that while bacterial symbionts may take a toll on their host’s energetic resources, and at times may even become pathogenic, they might also serve as immune-augmenting agents. The gregarious locusts, living at high densities with high exposure to pathogens, are therefore likely to harbor a more diverse microbiota, potentially supported by host-derived nutrients; whereas for solitary locusts, which experience lower exposure to pathogens, a simpler gut bacterial profile would suffice, imposing a lower energetic cost, albeit at greater risk of pathogenic infections [13]. This hypothesis is somewhat supported by the report of Wilson et al. [42], who found that gregarious locusts show higher pathogen resistance due to increased antimicrobial activity.

However, our recent study aimed at directly investigating the role of density per se on the bacterial composition residing within the hindgut of the desert locust did not reveal the same pattern of phase-dependent bacterial complexity [15]. Sequential sampling of laboratory-reared gregarious and solitary locusts revealed that while the solitary locust bacterial composition had remained roughly the same across the different sampling periods, the gregarious bacterial composition was either similar to that of the solitary locusts or, alternatively, taken-over by a dominant bacterial strain of *Weissella* sp., spreading within their dense population [15]. One possible explanation for the discrepancy between these findings and the findings described by Dillon et al. [13] for the brown locust’s bacterial profile (beyond genus-dependent difference), may lie in the locusts’ diet. While the laboratory insects of both phases were fed the exact same food, the field-caught gregarious and solitary locusts may have consumed different diets, particularly since gregarious and solitary locusts in the field tend to demonstrate different feeding patterns; with the gregarious diet being more diverse [1], driving their gut bacteria composition towards higher complexity, in accord with the findings of Dillon et al. [13]. Another possible explanation lies in the findings concerning the temporal changes in the gregarious bacterial composition. If the microbiota of the alimentary tract fluctuates across time, a sequential sampling protocol is needed in order to fully uncover the nature of the gut bacterial dynamics. Either way, it seems that while the locust gut bacterial composition may be affected by the different feeding patterns of gregarious and solitary locusts, the density-dependent phase per-se does not dictate the differences in the gut bacterial composition—meaning that there is no phase-dependent bacterial regulation by the host in the desert locust. These findings, of course, do not rule out the existence of a tentative density-dependent phase bacterial regulation mechanism in other locusts.

Continuous sampling and comparison of the bacterial composition in the spermatheca of laboratory-reared female desert locusts across several years have revealed a similar trend of occasional bacterial blooms in the gregarious females, while the solitary females maintained the same bacterial diversity across all sampling intervals [16]. These data suggest that while the locust’s bacterial composition may show no direct interactions with the locust density-dependent phase, it may nonetheless be affected indirectly by the different life histories of solitary and gregarious animals.

Recent new laboratory-collected data on the impact of a phase shift on the gut and integument microbiota indicate that upon joining a gregarious population, the gut bacterial composition of solitary *S. gregaria* undergoes a change to become similar to that of the gregarious phenotype, including the same *Weissella cibaria*-dominated composition as in the gregarious population [18]. This is in accord with the reported results regarding temporal shifts in the bacterial dynamics of gregarious lab populations [15]. *Weissella* is also widely abundant in the gut of gregarious L. *migratioria* [14,22,23], and was found to induce aggregation in nymphs of the German cockroach [43]. This suggests that the *W. cibaria* linkage to gregarious locusts may be stronger than previously considered, and may imply it has a colonization advantage in dense populations. Furthermore, this also raises the possibility that *Weissella* may even act as a crowding-promoting bacterium, contributing to the swarm’s integrity [18].

## 6. Concluding Remarks and Future Directions

During the last few decades, the microbiome of insects has been studied thoroughly in a variety of agriculturally and ecologically important species, with the aim of deciphering some of the complex, molecular mechanisms that may be involved in regulating the insect–host biology [20,30,44,45,46]. Despite several key discoveries made to date regarding the microbiome of the desert and the migrating locusts, many unanswered questions remain related to the bacterial residents of locusts and to host–microbe interactions. This is especially important in light of locusts being a devastating agricultural insect pest that performs large-scale migrations, and consequently has the ability to both acquire and disseminate microbial agents across vast and different habitats.

Open questions include the type and nature of bacteria residing within other locust species. Details of the interactions between the locust population dynamics and that of their bacterial symbionts are also missing. Specifically, the mechanisms by which a possible aggregation-promoting bacterium, such as *Weissella*, can drive locust populations towards gregariousness and maintain this state. While the bacterial symbionts in solitary locust field populations are far from fully described, a promising direction is establishing the role of the microbiome in driving phase change and swarming. Revealing such mechanisms may bring us closer to utilizing bacteria or other microbial agents to reduce aggregation and migration during locust eruptions, and thereby also potentially reduce their destructive impact.

Yet another largely uninvestigated—but important—aspect of locust–microbe interactions, is that of the locust’s symbiosis with fungi and viruses. Though some entomopathogenic fungi have been studied in the context of locust control [47], the fungal and viral compositions of locusts have been largely ignored, despite constituting an important unknown factor in our understanding of locust biology. These possible symbiotic micro-organisms harbor the potential to interact directly or indirectly with the host through other microbial organisms, to affect locust behavior, fitness, aggregation, and even pesticide resistance. Therefore, characterizing the composition of these microorganisms, and the nature of their symbiotic relations with the locust, will fill in an important gap in our knowledge of these fascinating insects.
